# Intermittent High Glucose Elevates Nuclear Localization of EZH2 to Cause H3K27me3-Dependent Repression of KLF2 Leading to Endothelial Inflammation

**DOI:** 10.3390/cells10102548

**Published:** 2021-09-26

**Authors:** Sumukh Thakar, Yash T Katakia, Shyam Kumar Ramakrishnan, Niyati Pandya Thakkar, Syamantak Majumder

**Affiliations:** Department of Biological Sciences, Birla Institute of Technology and Science (BITS) Pilani, Pilani Campus, Pilani 333 031, Rajasthan, India; p20170437@pilani.bits-pilani.ac.in (S.T.); yash.katakia@pilani.bits-pilani.ac.in (Y.T.K.); s.ramakrishnan@pilani.bits-pilani.ac.in (S.K.R.); niyati.pandya@pilani.bits-pilani.ac.in (N.P.T.)

**Keywords:** endothelial cells, enhancer of zeste homolog 2, epigenetics, H3K27me3, inflammation, KLF2

## Abstract

Epigenetic mechanisms have emerged as one of the key pathways promoting diabetes-associated complications. Herein, we explored the role of enhancer of zeste homolog 2 (EZH2) and its product histone 3 lysine 27 trimethylation (H3K27me3) in high glucose-mediated endothelial inflammation. To examine this, we treated cultured primary endothelial cells (EC) with different treatment conditions—namely, constant or intermittent or transient high glucose. Intermittent high glucose maximally induced endothelial inflammation by upregulating transcript and/or protein-level expression of ICAM1 and P-selectin and downregulating eNOS, KLF2, and KLF4 protein levels. We next investigated the underlining epigenetic mechanisms responsible for intermittent hyperglycemia-dependent endothelial inflammation. Compared with other high glucose treatment groups, intermittent high glucose-exposed EC exhibited an increased level of H3K27me3 caused by reduction in EZH2 threonine 367 phosphorylation and nuclear retention of EZH2. Intermittent high glucose also promoted polycomb repressive complex-2 (PRC2) assembly and EZH2′s recruitment to histone H3. Abrupt enrichment of H3K27me3 on *KLF2* and *KLF4* gene promoters caused repression of these genes, further supporting endothelial inflammation. In contrast, reducing H3K27me3 through small molecule and/or siRNA-mediated inhibition of EZH2 rescued KLF2 level and inhibited endothelial inflammation in intermittent high glucose-challenged cultured EC and isolated rat aorta. These findings indicate that abrupt chromatin modifications cause high glucose-dependent inflammatory switch of EC.

## 1. Introduction

The vascular endothelium maintains homeostasis by acting as a cellular lining of the circulatory system, and dysfunction of these cells leads to many pathophysiologies, including vasoconstriction and atherosclerosis [1]. Endothelial dysfunction is a common finding in patients affected by diabetes and hyperglycemic conditions that damage the endothelial lining of blood vessels, causing vascular complications [2]. Such dysfunction of the endothelium upon hyperglycemia is primarily manifested by a reduction in protective genes and expression of inflammation-associated genes in endothelial cells (EC) [3]. 

Hyperglycemic conditions in type 2 diabetes have been shown to cause epigenetic disturbances in many tissue types, including pancreatic islets, adipose tissue, skeletal muscle, and vascular tissues [4,5,6,7,8]. EC exposed to hyperglycemia exhibited heightened enrichment of di- and tri-methylated histone H3 lysine4 (H3K4me2/3) on the MCP-1 promoter [9]. Such an increase in H3K4me2/3 was due to an increase in the expression level of histone methyltransferase MLL3, menin, and SET7, along with a concomitant reduction in the demethylase LSD1. In parallel, Set7 was found to stimulate nuclear factor kappa-light-chain-enhancer of activated B cells (NF-κB)-dependent oxidative and inflammatory signaling by mono-methylation of H3K4 at its promoter in human aortic endothelial cells [10]. In an independent study, H3K9-specific demethylase JHDM2A (also known as KDM3A) was shown to be involved in regulating the expression of metabolic genes, indicating epigenetic regulation as key to diabetes-dependent microvascular complications [11]. The work of El-Osta et al. (2008) demonstrated a dynamic and coordinated alteration in H3K9/K14 acetylation and methylation of histones H3K4 that was responsible for p65-dependent inflammatory switch of EC [12]. Interestingly, the hyperglycemia-driven induction of gene expression that is responsible for endothelial dysfunction occurred through inversely modulating the acetylation of H3K9/K14 and methyl-CpG content of DNA [13]. Histone methyltransferase EZH2 is the enzymatic subunit of PRC2 and is primarily responsible for catalysis of H3K27me3, which finally executes PRC2-dependent gene repression through heterochromatinization [14]. EZH2 and its product H3K27me3 are critical in regulating signaling pathways responsible for development and diseases. In a recent study, human umbilical vein EC isolated from gestational diabetes mellitus-affected individuals (also referred as GDM-HUVECs) displayed impaired endothelial function via miR-101 upregulation and a concurrent reduction in EZH2 and H3K27me3 levels [15]. Although, said study described the role of EZH2 in maintaining endothelial homeostasis and function, it failed to narrate the exact underlying mechanisms through which EZH2 engendered an inflammatory state of EC upon high glucose challenge. 

Previous studies established that intermittent or oscillating glucose levels, such as those detected in diabetic individuals, have more deleterious effects than constant high glucose has on endothelial function and oxidative stress [16,17]. Intermittent high glucose exposure to HUVEC caused activation of protein kinase C and NAD(P)H-oxidase, leading to oxidative stress and further promotion of endothelial apoptosis [16,17]. Furthermore, individuals with type 2 diabetes showed heightened oxidative stress and impairment of endothelial function when challenged with an oscillating glucose level [16,17]. Therefore, altogether, these findings [16,17] suggest a stronger association between endothelial oxidative stress and dysfunction and intermittent/oscillating glucose level. Transient hyperglycemia induces long-lasting activation of epigenetic changes in the promoter of the nuclear factor κB (NF-κB) subunit p65 in aortic endothelial cells, both in vitro and in nondiabetic mice, which cause increased p65 gene expression [12]. A recent study by Flynn et al. described transient intermittent hyperglycemia as causing proliferation of monocytes by promoting myelopoiesis through the RAGE pathway in atherosclerosis [18]. Although inflammation-dependent epigenetic alteration upon hyperglycemic exposure is well studied in many cell types, the epigenetic mechanism that is responsible for high glucose-dependent endothelial inflammation is yet to be investigated. In this paper, we aimed to study the role of epigenetic regulation through EZH2–PRC2 pathways in intermittent high glucose-exposed EC and to further unravel the underlining mechanisms that facilitate inflammatory signaling in EC. 

## 2. Materials and Methods

### 2.1. Cell Culture and High Glucose Treatment Condition 

Human umbilical vein endothelial cells (HUVEC) were purchased from Hi Media (CL002-T25, Mumbai, India) and cultured using HiEndoXLTM Endothelial Cell Expansion medium (AL517, Hi Media) supplemented with 2% endothelial growth supplement, 5% fetal bovine serum (FBS), and 1% pen/strep. The HUVEC expansion medium contained 5.5 mM of glucose, and therefore HUVEC were grown in a medium containing 5.5 mM of basal glucose. EA.hy926, an immortalized human umbilical vein cell, kindly gifted by Dr. Suvro Chatterjee (Anna University, Chennai, India), was primarily used as human EC in this study. The cells were cultured and passaged every 2 or 3 days in Dulbecco’s modified Eagle’s medium (DMEM) (Hi Media Laboratories) supplemented with 10% FBS (Hi Media Laboratories) and 1% penicillin/streptomycin (Sigma-Aldrich, St. Louis, MO, USA). Both cells were cultured at 37 °C and in a humidified incubator with a 5% CO_2_ atmosphere. They were then trypsinized and seeded in six-well plates at a confluency of 1 × 10^5^. After achieving a 70% confluency, they were then subjected to different time kinetics of hyperglycemia, alternating between normal glucose level (5.5 mM) and high glucose level (25 mM); a constant high glucose (referred to as CHG) treatment condition was imparted on EC by incubating the cells for 72 h in high glucose (25 mM)-containing media; an intermittent high glucose (referred to as IHG) treatment condition was established through 12 h of high glucose (25 mM) and 12 h of normal glucose (5.5 mM) cycle for 3 cycles, totaling 72 h of treatment time; a transient high glucose (referred to as THG) treatment condition was achieved through 24 h of high glucose (25 mM) followed by 48 h of normal glucose (5.5 mM) exposure. A control treatment condition (cells constantly exposed to normal glucose (5.5 mM)) is represented as “Ct” in multiple figures. 

### 2.2. Animal Dissection and Treatment Conditions

All experimental procedures for the rodent studies were approved by the Institutional Animal Ethics Committee of BITS Pilani, Pilani Campus. Male Wistar rats aged 12 to 16 weeks were selected for the ex vivo experiment. The rats had been fed on normal chow diet. They were anaesthetized, and dissection was then performed by a cut from the ventral end. Hearts and the aortas were perfused with PBS to remove any blood cells from the vessels. The primary aortas were then collected and the fatty tissue layers were removed, followed by cutting into cylindrical pieces measuring 2 mm lengthwise to obtain aortic rings. These rings were washed with PBS and further cultured in HiEndo endothelial cell medium in 24-well plates for the experiment. Prior to initiating any treatment condition, these aortic rings were allowed a 12 h incubation period in the culture medium containing 5.5 mM glucose. The aortic rings were subjected to an intermittent glucose treatment, as explained earlier (12 h alternating periods of normal (5.5 mM) and high glucose (25 mM) for three cycles), along with a combination of GSK126 (10 μM). After 3 days, the tissue fractions were homogenized, suspended in RIPA lysis buffer, and sonicated. Protein estimation was done by Bradford assay, followed by SDS-PAGE and immunoblot. eNOS Rabbit mAb (1:500; #32027), ICAM1 pAb (1:500; #4915), EZH2 Rabbit mAb (1:1000; #5246), and H3K27me3 Rabbit mAb (1:1000; #9733) from Cell Signaling Technology (Danvers, MA, USA) and KLF2 Rabbit mAb (1:500, PA5-40591) from Thermo Fisher Scientific were used for probing.

### 2.3. Inhibitor Treatment Condition and RNA Silencing in Cultured Endothelial Cells 

Prevalidated human-specific EZH2 small-interfering RNA (SignalSilence^®^ Ezh2 siRNA I #6509) and control siRNA (SignalSilence^®^ Control siRNA#6568) were from Cell Signaling Technology. EZH2 siRNA was used at a concentration of 40 nM. OptiMEM containing lipofectamine 2000 (#11668030, Thermo Fisher Scientific, Waltham, MA, USA) and siRNA were subjected to HUVEC for 4 h. OptiMEM was later replaced with HiEndo endothelial culture medium and subjected to the intermittent high glucose (25 mM) treatment condition. Cells were finally harvested upon completion of the intermittent high glucose treatment condition, as specified earlier. For comparative analysis, all non-EZH2 siRNA-transfected HUVEC were transfected with scrambled siRNA. For all EZH2 inhibition studies, a competitive SAM inhibitor GSK126 was used at a final concentration of 10 μM. DMSO was used as vehicle control.

### 2.4. Cell Viability Assay

Viability of cells was measured by 3-(4,5-dimethylthiazol-2-yl)-2,5-di-phenylte- -trazolium bromide (MTT) assay. HUVEC were cultured in 96-well plates overnight, followed by exposing them to differential high glucose treatment conditions: a constant high glucose (25 mM) treatment condition for 72 h; two different conditions of intermittent high glucose treatment—(i) 8 h of high glucose (25 mM) and 16 h of normal glucose (5.5 mM) cycle for 3 cycles and (ii) 12 h of high glucose (25 mM) and 12 h of normal glucose (5.5 mM) cycle for 3 cycles; and two different conditions of transient high glucose treatment—(i) 4 h of high glucose (25 mM) followed by 68 h of normal glucose (5.5 mM) exposure and (ii) 24 h of high glucose (25 mM) followed by 48 h of normal glucose (5.5 mM) challenge. For GSK126 treatment groups, HUVEC were treated with GSK126 (10 μM) for 72 h. Thereafter, MTT was added to all treated and control cells, and cells were incubated for 4 h. Formazan crystals were solubilized in DMSO, and readings were obtained at 495 nm with a differential filter of 630 nm using a micro-plate reader (Start-fax 2100). Percentage of viable cells was calculated as % viability = (mean absorbance value of drug-treated cells)/(mean absorbance value of control) ∗ 100.

### 2.5. RNA Isolation

RNA isolation was performed following the manufacturer’s protocol (#15596026, TRIzol™ Reagent, Life Technologies, Thermo Fisher Scientific). In brief, HUVEC were grown in six-well plates up to 70% confluency and subjected to the earlier-mentioned treatment regimes. After 72 h, treated cells were incubated in Trizol reagent. Upon collecting the cells in Trizol reagent, organic layer separation was carried out using chloroform, and further RNA in the aqueous layer was precipitated using isopropanol. Next, precipitated RNA was washed with 75% ethanol, and RNA pellets were air dried. Finally, RNA pellets were dissolved in sterile nuclease-free water, and quantity and quality were analyzed through NanoDrop measurement.

### 2.6. cDNA Synthesis and Quantitative Analysis through Reverse Transcriptase-Quantitative Polymerase Chain Reaction

To measure the transcript level of different genes, we carried out reverse transcriptase-quantitative polymerase chain reaction (RT-qPCR). In brief, total RNA (1 μg) was taken from the cDNA preparation using iScript™ cDNA Synthesis Kit (#1708891, Bio-Rad Laboratories, Hercules, CA, USA). Prior to cDNA synthesis, isolated RNAs were pre-incubated with DNAse to remove any DNA contamination. Real-time PCR was then performed using iTaq™ Universal SYBR^®^ Green Supermix (#1725124, Bio-Rad Laboratories), with a total master mix volume of 10 μL. Analysis was carried out by calculating delta-delta Ct. GAPDH was used as the housekeeping gene. 

### 2.7. Subcellular Fractionation

HUVEC challenged with or without intermittent hyperglycemia, were harvested and washed with PBS. Upon centrifugation, the cell pellet was re-suspended in ice cold PBS containing 0.1% Nonidet P-40 (NP-40) and 1% protease inhibitors (#P8340, Sigma-Aldrich, MI, USA). The nuclear fraction was pelleted down by centrifugation for 10 min at 10,000 rpm, while the supernatant was collected as the membrane and cytoplasmic fraction. Furthermore, the nuclear lysate was obtained by resuspending the nuclear pellet in PBS containing 0.1% Nonidet P-40 (NP-40) and 1% protease inhibitor (#P8340, Sigma). Both the nuclear and cellular fractions were sonicated for 10 s (×2) and then separated by SDS-PAGE and immunoblotted.

### 2.8. Co-Immunoprecipitation

Proteins were extracted from HUVEC using RIPA lysis buffer (#R0278, Sigma-Aldrich) containing protease inhibitor (#P8340, Sigma-Aldrich). A total of 1000 μg of protein was used for pulldown with the antibody for EZH2 (1 μg/uL, #5246, Cell Signaling Technology), followed by separating in SDS-PAGE. Antibodies directed to SUZ12 (1:1000, #3737), EED (1:1000, #51673), and histone H3 (1:1000, #4499) (Cell Signaling Technology) were used to confirm co-immunoprecipitation with EZH2. Resulting bands were visualized by ChemiDoc using the Clarity™ (#1705061, Bio-Rad Laboratories) or Clarity™ Max (#1705062, Bio-Rad Laboratories) Western Blotting ECL Substrates and analyzed by expression levels relative to 5% input obtained using ImageJ software.

### 2.9. Immunoblotting 

HUVEC were grown up to 70% confluency for high glucose treatment conditions. Medium was removed and cells were briefly washed with sterile 1X phosphate-buffered saline (PBS). RIPA buffer containing protease inhibitor was then used for protein extraction. Cells were incubated in RIPA buffer for 1 h, followed by sonication. Cell lysates were centrifuged (10,000 rpm, 8 min), followed by collecting the supernatant and measuring protein concentration using Bradford reagent. Equal amounts of protein for different treatment groups were then applied in SDS-PAGE, followed by transferring the proteins onto a nitrocellulose membrane. Membranes were then blocked in 5% nonfat milk or 5% bovine serum albumin for 1 h. Membranes were then probed overnight at 4 °C with different primary antibodies: eNOS (1:1000, #32027), ICAM1 (1:1000, #4915), EZH2 (1:1000, #5246), H3K27me3(1:1000,#9733), UTX Rabbit mAb (1:1000; #33510), GAPDH Rabbit mAb (1:1000, #5174), KLF2 Rabbit pAb (1:1000), KLF4 Rabbit mAb (1:1000), H3 Mouse mAb (1:2000, #4499) (Cell Signaling Technology), and Phospho- EZH2 (Thr367) Rabbit pAb (1:1000; #PA5-106225, Thermo Fisher Scientific). The blots were then incubated with a peroxidase-conjugated anti-rabbit or anti-mouse IgG antibody (1:2000) (#7074 or #7076, respectively; Cell Signaling Technology). The antibody–antigen reactions were detected using the Clarity™ or Clarity™ Max Western Blotting ECL Substrates (Bio-Rad). Densitometry analysis was performed using ImageJ software.

### 2.10. Immunofluorescence Imaging and Analysis

HUVEC were grown on gelatin (#TC041, HiMedia)-coated coverslips up to 70% confluency. After completion of treatment, they were washed with PBS and subjected to fixation with 4% ice-cold paraformaldehyde. Cells were incubated with 0.1% Triton X for permeabilization. Cells were then incubated with BSA (1%) for 1 h, followed by overnight incubation with EZH2 antibody (1:1000, #5246, Cell Signaling Technology). Cells were washed with PBS to remove unbound primary antibody, and were subsequently incubated with Alexa fluor 555 secondary antibody (1:4000; #A32732, Thermo Fisher Scientific) for 2 h. To stain F-actin, cells were incubated with rhodamine-tagged phalloidin (1:5000; #R415, Thermo Fisher Scientific) for 30 min, followed by incubation with DAPI (#D9542, Sigma-Aldrich) for nuclear staining. Fluorescence images were captured using a Zeiss ApoTome.2 microscope (Carl Zeiss, Jena, Germany), and intensities were measured using ImageJ software.

### 2.11. Chromatin Immunoprecipitation (ChIP) and Subsequent Quantitative PCR

A ChIP assay was performed using an Imprint^®^ Chromatin Immunoprecipitation Kit (#CHP1, Sigma-Aldrich). In brief, exponentially growing HUVEC (80% cell density) were subjected to intermittent high glucose treatment. Treated HUVEC were harvested (1 × 10^6^ cells), washed, and cross-linked with 1% formaldehyde in HiEndoXLTM Endothelial Cell Expansion medium (10 min at room temperature). After washing in PBS, the cell pellet was re-suspended in Nuclei Preparation Buffer (200 µL per 10^6^ cells) and kept on ice for 10 min. The nuclear pellet thus obtained was re-suspended in shearing buffer (100 µL per 10^6^ cells) supplemented with protease inhibitor cocktail (1 µL per ml of shearing buffer) and further sheared by sonication for 30 s (×40). The sheared chromatin (containing 100 to 500 bp long sheared genomic DNA) was immunoprecipitated with antibodies directed against H3K27me3 at a concentration of 1:50 (#9733, Cell Signaling Technology). The samples were then washed, reverse cross-linked, and treated with proteinase K to obtain purified DNA fragments [19]. qPCR was performed using primers targeted to amplify regions of human *KLF2* and *KLF4* gene promoters. 

### 2.12. Primer Sequences for Transcript and Promoter Primers

Primer sequences to analyze the transcript level of different genes and ChIP-qPCR primer to analyze promoter level enrichment of H3K27me3 are provided below in Table 1. 

### 2.13. Statistics

All the values are expressed as the mean ± SD. All analysis data in bar graphs are presented as relative to control treatment condition. Statistical significance was determined by one-way ANOVA followed by Fisher least significant difference post hoc test for multiple groups comparison or by two-tailed Student’s *t* test for comparison between two groups, unless otherwise stated. Statistical analyses were performed using GraphPad Prism software. A *p* value of less than 0.05 was considered statistically significant. 

## 3. Results

### 3.1. Intermittent High Glucose Treatment Maximally Induced Endothelial Inflammation In Vitro and Ex Vivo

Intermittent or oscillating glucose causes more deleterious effects on endothelial function than constant high glucose, especially on its oxidative balance [16,17]. Therefore, we first undertook experiments to evaluate the effectivity of intermittent high glucose in inducing endothelial inflammatory signaling compared with constant or transient high glucose treatment conditions. Initially, to determine the treatment conditions, we assessed the viability of HUVEC that were exposed to differential high glucose treatment conditions. Although subtle changes in cell viability were detected in some of the high glucose treatment conditions, such differences were statistically insignificant (Supplemental Figure S1A). To analyze endothelial inflammation, we then measured the transcript level of endothelial inflammatory cell adhesion molecule ICAM1 in cells exposed to the differential high glucose challenge. Cells in the intermittent high glucose treatment condition, where HUVEC were incubated in 12 h high and normal glucose cycles, exhibited the highest transcript-level expression of ICAM1 (Supplemental Figure S1B). 

**Figure 1:**
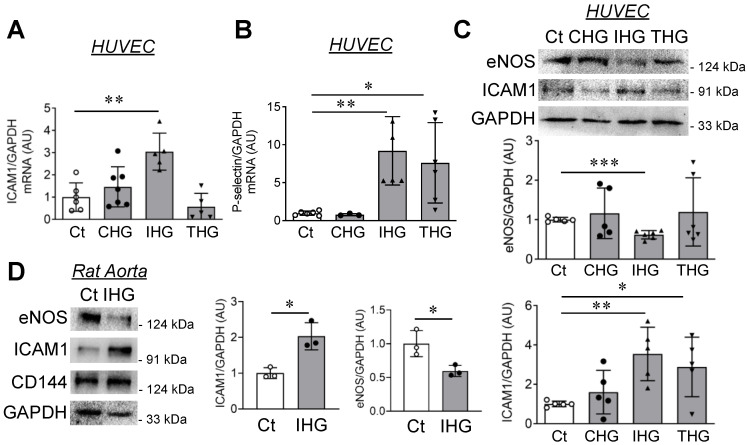
Intermittent hyperglycemia triggers an inflammatory state of endothelial cells. (**A**,**B**) Transcriptomic analysis of ICAM1 ((**A**), *n* = 5) and P-selectin (**B**), *n* = 4–6) in HUVEC exposed to differential high glucose treatment conditions; control, constant (72 h high glucose, 25 mM), intermittent (alternating 5.5 mM and 25 mM cycle of 12 h for 3 cycles), and transient (24 h high glucose, 25 mM, followed by 48 h of normal glucose, 5.5 mM). (**C**) Immunoblotting and quantitation for eNOS and ICAM1 in HUVEC challenged with previously mentioned high glucose treatment conditions (*n* = 5–6). (**D**) Immunoblotting and quantitation of eNOS, ICAM1, and CD144 in tissue lysates of rat aortic rings (2 mm length) that were exposed to intermittent high glucose (*n* = 3). Control treatment condition (cells constantly exposed to normal glucose (5.5 mM)) represented as “Ct” in the figure. All analyzed data were normalized to the control treatment condition. Values represent the mean ± SD. * *p* < 0.05, ** *p* < 0.01 and *** *p* < 0.001 by one-way ANOVA analysis or by unpaired *t* test.

Based on these observations, we next shortlisted three unique high glucose treatment regimens—constant high glucose (72 h of constant high glucose), intermittent high glucose (12 h high and normal glucose cycles for three cycles), and transient high glucose (24 h high glucose followed by 48 h of normal glucose)—for comprehensive analysis of endothelial inflammatory markers. Transcript-level expression of ICAM1, VCAM1, and P-selectin were carried out. A significant increase in the transcript level of ICAM1 and P-selectin were detected in cells exposed to intermittent high glucose, while no changes in ICAM1 were detected in constant or transient high glucose-treated cells (Figure 1A,B). We did not detect any changes in VCAM1 transcript level in any of the treatment conditions (data not shown). We then measured the protein-level expression of ICAM1 and eNOS and further observed a significant increase in ICAM1 expression and reduction in eNOS level in cells treated with intermittent high glucose, while no changes in these proteins were detected in EC exposed to constant or transient high glucose relative to normal glucose-incubated HUVEC (Figure 1C). To exclude the possibility that the intermittent high glucose effect on endothelial inflammation was due to osmolality changes, we simultaneously analyzed these markers in HUVEC exposed to intermittent mannitol, which generates a comparable osmolality that is similar to intermittent high glucose. We did not observe any alteration in eNOS (Supplemental Figure S2A) and ICAM1 (Supplemental Figure S2B) protein level in EC challenged with intermittent mannitol. 

To confirm such in vitro findings in an animal model, we isolated rat aortas and exposed them to the intermittent high glucose treatment condition ex vivo. Through such experiments, we observed a reduction in eNOS level and a concurrent increase in ICAM1 expression in aortas exposed to intermittent high glucose (Figure 1D). We also confirmed the presence of EC in the rat aortic tissue lysate by detecting the presence of CD144, an endothelial-specific cell-surface marker (Figure 1D). 

### 3.2. Intermittent High Glucose Causes Nuclear Localization of EZH2 through Its Threonine 367 Phosphorylation, Thereby Elevating H3K27me3 Level 

**Figure 2:**
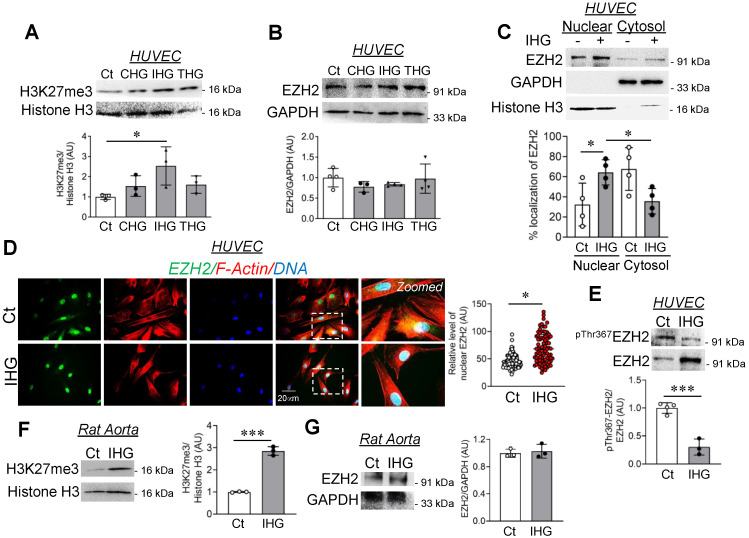
Intermittent high glucose induces nuclear localization of EZH2 through de-phosphorylation of threonine 367. (**A**,**B**) Immunoblotting and quantitation of differential hyperglycemia-treated HUVEC lysates for H3K27me3 ((**A**), *n* = 3) and EZH2 ((**B**), *n* = 4). (**C**) Subcellular fractionation, immunoblotting, and quantitation of nuclear and cytosolic level of EZH2 in intermittent high glucose-challenged HUVEC (*n* = 4). (**D**) Immunofluorescence and quantitation of HUVEC for EZH2 (green) after intermittent high glucose treatment. F-actin staining through phalloidin shown in red and DAPI staining shown in blue. Fluorescence intensity values taken for individual cells are each indicated by dots, and at least *n* > 100 cells were analyzed per group from three independent experiments. (**E**) Immunoblotting and quantitation of threonine 367 phosphorylated EZH2 in intermittent high glucose-exposed HUVEC (*n* = 4). (**F**,**G**) Tissue lysates of intermittent high glucose-treated rat aortic rings were immunoblotted and quantified for H3K27me3 (**F**) and EZH2 (**G**) (*n* = 3). Control treatment condition (cells constantly exposed to normal glucose (5.5 mM)) represented as “Ct” in the figure. All analyzed data were normalized to the control treatment condition. Values represent the mean ± SD. * *p* < 0.05 and *** *p* < 0.001 by unpaired *t* test.

Once we established that intermittent high glucose was most potent in causing the inflammatory switch of EC, we next undertook experiments to evaluate the underlining epigenetic mechanisms. Alterations in epigenetic mechanisms are prevalent in different cells exposed to hyperglycemic conditions. In a recent study, hyperglycemia was shown to promote *SIRT6* and *TETs*, in turn causing dynamic changes in 5 methyl cytosine and 5 hydroxy methyl cytosine in WBCs collected from diabetes mellitus type 2 (T2DM) patients [20]. In addition, EC exposed to constant hyperglycemia exhibited heightened enrichment of di- and tri-methylated histone H3 lysine4 (H3K4me2/3) on MCP-1 promotor [9] through histone methyltransferase MLL3, menin, and SET7. We therefore questioned whether intermittent high glucose alters the epigenetic landscape of EC through regulation of EZH2 and its product, H3K27me3. Immunoblot analysis of H3K27me3 and EZH2 in HUVEC treated with different high glucose treatment conditions revealed increased H3K27me3 in cells exposed to intermittent high glucose (Figure 2A). Surprisingly, we did not detect any changes in the methyltransferase EZH2 (Figure 2B)- or H3K27me3-associated demethylase UTX (Supplemental Figure S3A,B) and JMJD3 (Supplemental Figure S3C,D) in EC exposed to intermittent high glucose. Unlike intermittent high glucose, intermittent mannitol treatment (considered as osmolality control) in EC did not alter cellular H3K27me3 levels (Supplemental Figure S2C). 

Considering that EZH2 having a dispersive localization within cells and that its cytosolic localization was shown to execute certain cytosolic functions [21], we further examined the localization of EZH2 protein in EC exposed to intermittent high glucose. Intermittent high glucose treatment enhanced the nuclear localization of EZH2 in EC (Figure 2C,D). As the studies up to now describe that p38-dependent phosphorylation of EZH2 protein at threonine 367 (^pThr367^EZH2) causes its cytosolic localization [22], we determined the level of ^pThr367^EZH2 in EC exposed to intermittent high glucose. Through such analysis, we detected a significant reduction in endothelial ^pThr367^EZH2 upon intermittent high glucose treatment (Figure 2E), thereby allowing EZH2′s nuclear translocation in EC upon such treatment condition. Furthermore, we next confirmed these in vitro observations in an ex vivo model of rat aortic rings, which indicated a robust increase in H3K27me3 (Figure 2F) level without altering the expression level of EZH2 (Figure 2G) upon intermittent high glucose exposure. 

### 3.3. In Endothelial Cells Treated with Intermittent High Glucose, Nuclear EZH2 Assembles PRC2 That Endorses H3K27me3 Enrichment on KLF2 and KLF4 Promoters and Further Suppresses Their Expression 

**Figure 3:**
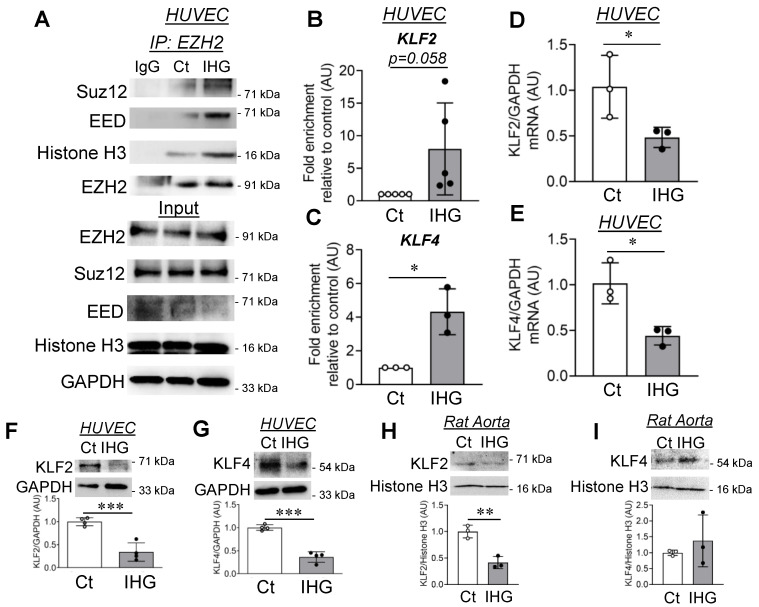
Intermittent hyperglycemia causes EZH2 coupling with PRC2 proteins, leading to H3K27 trimethylation at promoter regions of the *KLF2* and *KLF4* genes. (**A**) Co-immunoprecipitation with EZH2 antibody, followed by immunoblotting for Suz12, EED, and histone H3 in both immuno-precipitated and total cell lysate (input) sample (*n* = 3). (**B**,**C**) ChIP assay using H3K27me3 antibody, followed by qPCR using primers specific to amplify promoter regions of *KLF2* gene ((**B**), *n* = 5) and *KLF4* gene ((**C**), *n* = 3) in HUVEC cells treated with intermittent high glucose. (**D**,**E**) Transcriptomic quantitation of *KLF2* ((**D**), *n* = 3) and *KLF4* ((**E**), *n* = 3) in HUVEC challenged with intermittent high glucose. (**F**,**G**) Immunoblotting and quantitation for KLF2 ((**F**), *n* = 4) and KLF4 ((**G**), *n* = 4) in intermittent high glucose-challenged HUVEC. (**H**,**I**). Tissue lysates of intermittent high glucose-treated rat aortic rings were immunoblotted for KLF2 ((**H**), *n* = 3) and KLF4 ((**I**), *n* = 3). Control treatment condition (cells constantly exposed to normal glucose (5.5 mM)) represented as “Ct” in the figure. All analyzed data were normalized to the control treatment condition. Values represent the mean ± SD. * *p* < 0.05, ** *p* < 0.01, and *** *p* < 0.001 by unpaired *t* test.

EZH2, along with other proteins including EED and Suz12, assembles PRC2 that catalyzes the H3K27me3 [23]. As an increase in the level of nuclear EZH2 was detected in EC exposed to intermittent high glucose, we wondered whether such elevated nuclear EZH2 assembles PRC2 to cause gene-specific H3K27me3 enrichment. To address this, we performed a co-immunoprecipitation experiment to evaluate the assembly of the PRC2 complex. By doing so, we detected a greater association of EED and Suz12 with EZH2 protein upon intermittent high glucose exposure (Figure 3A). Furthermore, in intermittent high glucose-treated EC, EZH2 was also found to be robustly associated with histone H3 (Figure 3A). Immunoblotting of total cell lysate revealed unaltered protein expression of EED and Suz12 upon intermittent high glucose exposure (Figure 3A, input panel). 

Transcription factors KLF2 [24,25] and KLF4 [26] are essential for maintaining endothelial homeostasis [27] and coordinate a gene expression pattern that inhibits the inflammatory switch of EC. Because we observed an elevated level of endothelial inflammation and concurrent increase in H3K27me3 level, we therefore questioned whether such increase in H3K27me3 may affect *KLF2* and *KLF4* genes through regulation of their corresponding gene promoter regions. To address this, we then performed a ChIP-qPCR assay to confirm the enrichment of H3K27me3 in *KLF2* and *KLF4* gene promoters in intermittent high glucose-exposed EC. We detected a more than 8-fold enrichment (although such increase in enrichment was found to be statistically insignificant due to large variation between the replicates, *p* = 0.058) of H3K27me3 at the *KLF2* gene promoter in intermittent high glucose-treated EC (Figure 3B), while the same treatment condition caused a nearly 5-fold increase in H3K27me3 enrichment at the *KLF4* gene promoter region (Figure 3C).

Since we observed elevated promoter-level enrichment of H3K27me3 on *KLF2* and *KLF4* gene promoters in EC challenged with intermittent high glucose, we next detected the transcript and protein-level expression of KLF2 and KLF4. We detected significant depletion in *KLF2* (Figure 3D) and *KLF4* (Figure 3E) mRNA in EC exposed to intermittent high glucose. Further, we observed that intermittent high glucose exposure led to a significant reduction in KLF2 (Figure 3F and Figure S3) and KLF4 (Figure 3G and Figure S4) protein levels. We did not detect any changes in KLF2 (Supplemental Figure S2A) and KLF4 (Supplemental Figure S2B) proteins in EC exposed to intermittent mannitol treatment. We also determined the expression level of KLF2 and KLF4 in tissue lysates collected from intermittent high glucose-challenged rat aortic rings and detected a significant reduction in KLF2 protein level (Figure 3H) and an unaltered KLF4 protein level (Figure 3I). Such observation of unaltered KLF4 protein level in intermittent high glucose-exposed rat aortic rings could likely be due to the complexity of the tissue and with the presence of other cells types, including smooth muscle cells that predominantly express KLF4 [28]. 

### 3.4. Inhibition of EZH2′s Methyltransferase Activity or siRNA-Mediated Knockdown of EZH2 Reverses Intermittent High Glucose-Dependent Endothelial Inflammation

**Figure 4:**
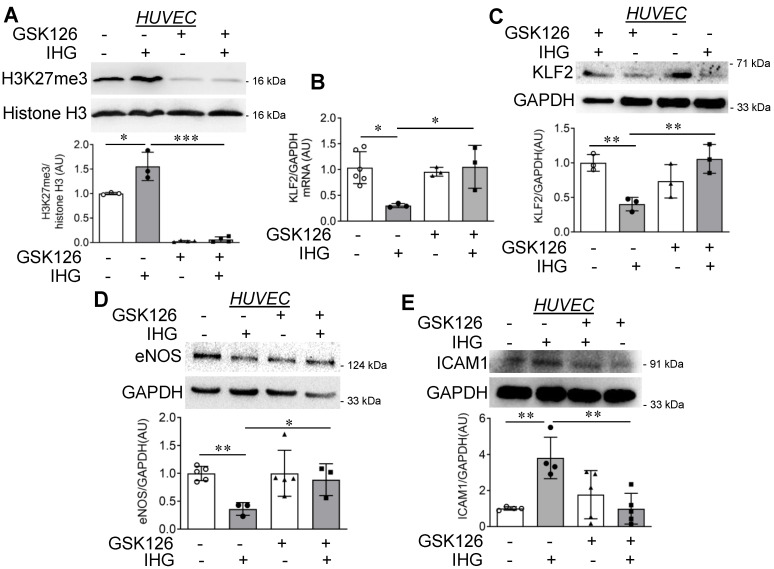
Small molecule-mediated inhibition of EZH2 in intermittent high glucose-exposed HUVEC decreases H3K27me3 level and blocks the inflammatory switch of EC. (**A**) Immunoblotting for H3K27me3 (*n* = 3) in GSK126-treated and intermittent high glucose-exposed HUVEC. (**B**) *KLF2* mRNA (*n* = 3) expression in HUVEC challenged with intermittent high glucose (alternating 5.5 mM (normal glucose) and 25 mM (high glucose) cycle of 12 h for 3 cycles) in combination with GSK126 (10 μM for 72 h). (**C**–**E**) KLF2 (**C**), eNOS (**D**), and ICAM1 (**E**) in cultured HUVEC treated with the EZH2 inhibitor GSK126 (10 μM for 72 h) in combination with intermittent high glucose (*n* = 3). Cells continuously exposed to normal glucose (5.5 mM) were considered as the non-intermittent high glucose treatment condition. All analyzed data were normalized to the control treatment condition. Values represent the mean ± SD. * *p* < 0.05, ** *p* < 0.01, and *** *p* < 0.001 by unpaired *t* test.

Because we established that nuclear EZH2 assembles PRC2 to cause an H3K27me3-dependent decrease in KLF2 and KLF4 level, we therefore investigated whether inhibition of EZH2′s methyltransferase activity reverses the endothelial inflammatory phenotype upon intermittent high glucose exposure. To explore this possibility, we took advantage of a selective inhibitor of EZH2, GSK126. We detected no change in viability of HUVEC that were exposed to 10 μM of GSK126 (Supplemental Figure S5A). To address the non-specific effect of GSK126, we also performed a comprehensive analysis of GSK126 effects on global histone H3 tri-methylation at different locations—lysine 4, lysine 9, lysine 27, lysine 36, and lysine 79—to understand its non-specific effect on different methyltransferases catalyzing tri-methylation in these specific sites. With EZH2, being the specific target of GSK126, we observed a significant reduction in H3K27me3, while the level of H3K4me3, H3K9me3, H3K26me3, and H3K79me3 majorly remained unaltered in both HUVEC (Supplemental Figure S5B–F) and HUVEC-derived transformed cell line EA.hy926 cells (Supplemental Figure S5G–K). GSK126 exposure further caused a complete inhibition of H3K27me3 content in HUVEC exposed to intermittent high glucose (Figure 4A). Coincident with the reduction in H3K27me3 levels, *KLF2* mRNA (Figure 4B) and protein level (Figure 4C) were elevated in HUVEC that were co-treated with intermittent high glucose and GSK126 combination in comparison with only the intermittent high glucose-treated group. Correspondingly, in the presence of GSK126, intermittent high glucose exposure failed to diminish eNOS protein level (Figure 4D) and to increase in ICAM1 protein level (Figure 4E).

**Figure 5:**
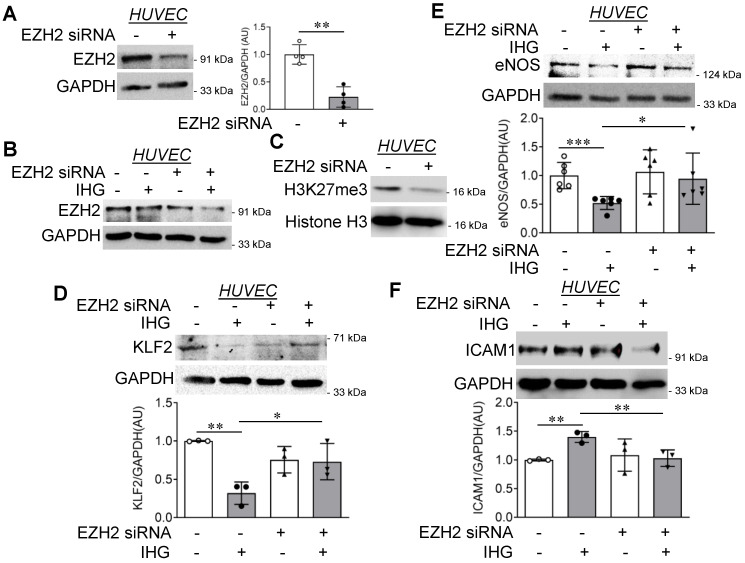
EZH2 knockdown in HUVEC reduces H3K27me3 and reverses intermittent high glucose-driven endothelial inflammation. (**A**–**E**) Immunoblotting of EZH2 in normal glucose (5.5 mM)-treated HUVEC ((**A**), *n* = 4), EZH2 in both normal glucose (5.5 mM) and intermittent high glucose-treated HUVEC ((**B**), *n* = 3), H3K27me3 ((**C**), *n* = 3) and quantification of KLF2 ((**D**), *n* = 3), eNOS ((**E**), *n* = 6), and ICAM1 ((**F**), *n* = 3) in HUVEC cells transiently transfected with scrambled or EZH2 siRNA, followed by challenging with intermittent high glucose. Cells continuously exposed to normal glucose (5.5 mM) were considered as the non-intermittent high glucose treatment condition. All analyzed data were normalized to the control treatment condition. Values represent the mean ± SD. * *p* < 0.05, ** *p* < 0.01, and *** *p* < 0.001 by unpaired *t* test.

Next, we took an alternative molecular approach to inhibit EZH2 by using specific siRNA and studied the inflammatory effect of intermittent high glucose on HUVEC with diminished EZH2 level. A time course experiment to measure the time-dependent reduction in EZH2 transcript and protein level exhibited a gradual decrease in EZH2 transcript and protein level in EA.hy926 cells and HUVEC, respectively, that were transfected with EZH2 siRNA for 24, 48, and 72 h (Supplemental Table S1 and Supplemental Figure S6A). We then detected a significant reduction in EZH2 protein level (Figure 5A) upon EZH2-specific siRNA transfection, with a concurrent reduction in H3K27me3 (Figure 5C). Such reduction in EZH2 protein level was also apparent in cells that were exposed to intermittent hyperglycemia (Figure 5B). Furthermore, intermittent high glucose-dependent reduction in KLF2 (Figure 5C) and eNOS (Figure 5D) protein levels were reversed in HUVEC transfected with EZH2 siRNA. In contrast, elevated ICAM1 protein level in intermittent high glucose-exposed cells was found to be diminished upon EZH2 knockdown (Figure 5E). In order to understand the effect of robust EZH2 inhibition through both the molecular and small molecule inhibitor approach, we next performed eNOS analysis in EZH2 siRNA-transfected and GSK126-treated HUVEC that were exposed to intermittent high glucose. We detected both GSK126 alone or EZH2 siRNA+GSK126 comparably (2.287-fold versus 2.317-fold relative to control) reversed eNOS protein level in HUVEC that were challenged with intermittent high glucose (Supplemental Figure S6B). 

### 3.5. Inhibition of EZH2 Blocks Intermittent High Glucose-Driven Endothelial Inflammation in Ex Vivo Rat Aortic Ring Model 

**Figure 6:**
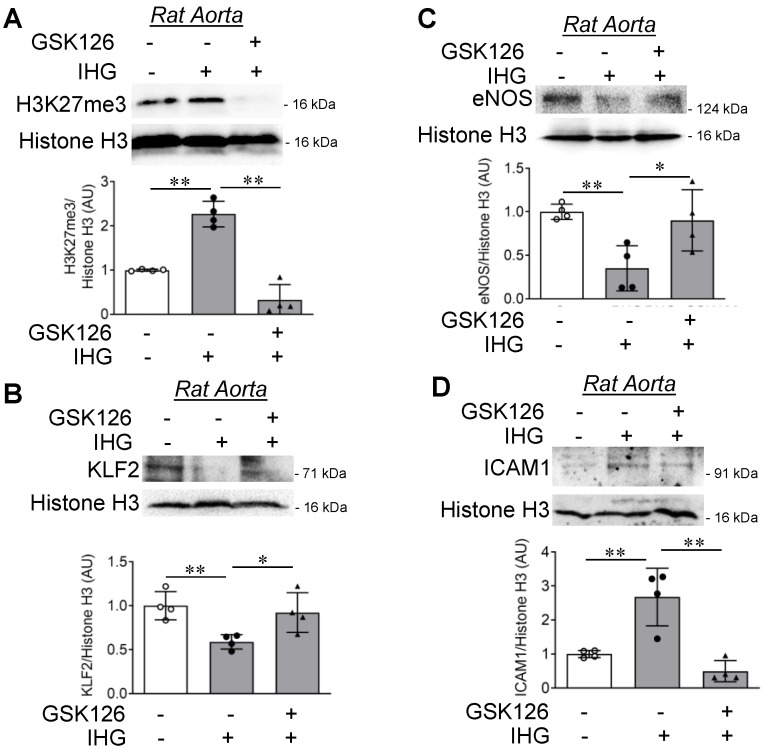
Inhibition of EZH2 in rat aortic rings exposed to intermittent hyperglycemia reduces H3K27me3 levels and blocks inflammatory signaling ex vivo. (**A**–**D**) Immunoblotting for H3K27me3 ((**A**), *n* = 4), KLF2 ((**B**), *n* = 4), eNOS ((**C**), *n* = 4), and ICAM1 ((**D**), *n* = 4) in rat aortic rings challenged with the EZH2 inhibitor GSK126 (10 μM for 72 h) in combination with intermittent high glucose. Tissues continuously exposed to normal glucose (5.5 mM) were considered as the non-intermittent high glucose treatment condition. All analyzed data were normalized to the control treatment condition. Values represent the mean ± SD. * *p* < 0.05 and ** *p* < 0.01 by unpaired *t* test.

Having found that intermittent high glucose promoted endothelial inflammation in cultured EC, while EZH2 inhibition or knockdown blocked changes in such inflammatory pathways, we next performed an EZH2 small molecule inhibition study to assess its efficacy in attenuating endothelial inflammation in rat aortic rings that were challenged with intermittent hyperglycemia. Treatment with GSK126 robustly inhibited an intermittent high glucose-dependent increase in H3K27me3 level (Figure 6A). Next, by measuring endothelial inflammatory markers in the treated rat aortic rings, we found that intermittent high glucose significantly abrogated total KLF2 (Figure 6B) and eNOS (Figure 6C) protein, while increasing ICAM1 level (Figure 6D). In contrast, when EZH2 was inhibited in these conditions, intermittent high glucose treatment failed to impart its effect on protein-level expression changes of KLF2, eNOS, and ICAM1 (Figure 6B–D). 

## 4. Discussion

**Figure 7:**
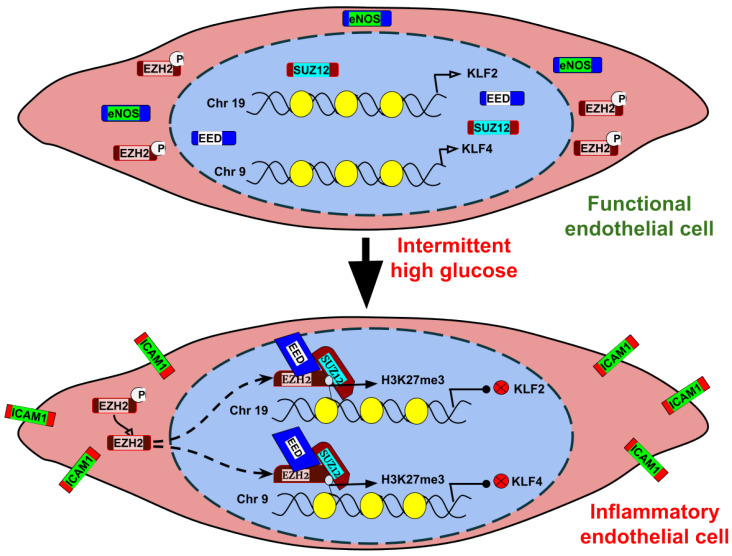
Mechanism by which elevated H3K27me3 level upon intermittent high glucose exposure promotes inflammatory signaling in endothelial cells. In normal human endothelial cells, the histone methyltransferase EZH2 localizes primarily in cytosol and thus causes restricted tri-methylation of lysine residue 27 (K27) on histone H3 (H3K27me3). During such condition, the expression levels of essential signaling molecules such as KLF2, KLF4, and eNOS are maintained in endothelial cells to allow physiological function. In human endothelial cells exposed to intermittent hyperglycemia, this causes nuclear localization of EZH2 by de-phosphorylating Thr367 phosphorylation. Once localized in the nucleus, it assembles PRC2 and binds to histone H3 to cause an elevated quantity of H3K27me3. Such gain of H3K27me3 upon intermittent hyperglycemic exposure results in repression of KLF2 and KLF4 through promoter-level enrichment of H3K27me3. Repression of KLF2 and KLF4 initiates inflammatory signaling in endothelial cells through downregulation of eNOS and upregulation of ICAM1.

Epigenetic regulation of gene expression has emerged as an essential mechanism for regulating endothelial function during development and in diseases. Post-translational modification of histone proteins is one the many epigenetic mechanisms that contribute to the differential gene expression pattern responsible for endothelial dysfunction. For instance, trimethylation of lysine residue 27 on histone H3 by methyltransferase EZH2 at the promoter region of target genes may lead to transcriptional silencing. EZH2-mediated regulation of gene expression through its H3K27me3 catalytic activity is well known to control the pathogenesis of different diseases; however, how such mechanisms drive endothelial inflammation and its dysfunction upon hyperglycemia challenge is not clearly understood. Here, we describe that intermittent high glucose exposure predominantly causes a robust inflammatory switch of EC through modulation of histone methylation. In specific, it alters the cellular level of H3K27me3 by promoting EZH2 nuclear localization and PRC2 assembly, followed by PRC2′s recruitment to histone H3. Such recruitment of PRC2 to histone H3 causes an abrupt enrichment of H3K27me3 in the gene promoters of two important transcription factors essential for endothelial function—KLF2 and KLF4—thereby causing their repression (Figure 7). Targeting EZH2 through small molecule inhibitors or siRNA-attenuated intermittent high glucose-mediated increase in H3K27me3 thus restores KLF2 level and reverses the inflammatory phenotype of EC. 

Diabetic individuals are at greater risk of developing vascular complications that further lead to cardiovascular diseases. Indeed, vascular complications associated with diabetes are manifested by early dysfunction and damage to the endothelial layer, suggesting that endothelial dysfunction is an early indicator of diabetes-associated complications [27]. Interestingly, individuals with type 2 diabetes mellitus show a unique pattern of glucose throughout the day. For instance, plasma glucose concentration is strictly controlled within a narrow range in normal subjects; however, diabetic individuals show plasma glucose level variation largely within a single day, outlined as “spikes” and “dips” [16]. Fluctuation in blood glucose level in people with mild to moderate hyperglycemia—following oral glucose load but not in the fasting state—is considered to be an ideal indicator of cardiovascular risk [29]. Moreover, there is growing evidence that an acute increase in glycemia (hyperglycemia spikes) and abrupt fluctuations in plasma glucose level more potently cause endothelial dysfunction [30]. Previously, intermittent high glucose induced a greater expression of the adhesion molecules, such as ICAM1, VCAM1, and E-selectin, in HUVEC than stable (constant) high glucose due to activation of PKCbeta and mitochondria-mediated oxidative stress [31]. In parallel with these observations, through our study, we determined that in comparison with constant or transient high glucose treatment conditions, intermittent high glucose exposure was prominent in inducing gene expression changes associated with endothelial inflammation. We also observed that such intermittent high glucose exposure caused a significant reduction in two important transcription factors—KLF2 and KLF4—that are essential for endothelial function and maintaining endothelial homeostasis [26]. Although previous findings reported elevation in ICAM1 and E-selectin expression in HUVEC upon constant high glucose exposure [32], previous reports and our current findings clearly indicate a stronger response to intermittent high glucose in enhancing the expression of these adhesion molecules. In addition, clinical studies in type 2 diabetic subjects established a strong correlation between oscillating blood glucose level and pathological cardiovascular events [16,17], indicating that oscillating/intermittent high glucose is more deleterious to the cardiovascular system. While a transient high glucose condition is reported to cause inflammatory signaling in EC [12], we also found in our current setting and condition that transient high glucose also caused a selective increase in certain adhesion molecules, such as P-selectin.

Hyperglycemia has been implicated in epigenetic modifications such as DNA methylation and histone modifications. Miao et al. showed that high glucose exposure led to increased H3K4me2 and H3K9me2 levels. They reported that the heightened levels of H3K4me2 were associated with increased methylation of nine genes, including ICAM3, FOS, GSTA-4, IL-8, and BCL-9—many of which are associated with inflammation [33]. Floris et al. isolated HUVEC from the umbilical cords collected from healthy or gestational diabetes mellitus-affected human subjects (referred to as GDM-HUVEC) and demonstrated a reduction in cellular H3K27me3 level due to concomitant reduction in EZH2 level via miR101 [15]. In contrast, we observed that intermittent high glucose exposure to HUVEC isolated from healthy individual (as specified by the supplier) elevated cellular H3K27me3 level and that such a hyperglycemic treatment condition preferentially favored an inflammatory state in endothelial cells. Such dissimilarity in the observation could be explained through the fact that in the previously reported work, Floris et al. measured the level of EZH2 and H3K27me3 in HUVEC that were directly harvested from individuals with gestational diabetes mellitus. In contrast, in the present study, EZH2 and H3K27me3 was measured in HUVEC (isolated from healthy human subjects) that were challenged with intermittent high glucose under a culture condition. Because oscillatory/intermittent blood glucose level through the day is apparent in people with type 2 diabetes [16,17], our study more robustly complements studies of such high glucose exposure under in vitro conditions. 

A coordinated alteration by histone methyltransferase SET7 and demethylase LSD1 dynamically alters H3K4 mono-methylation and H3K9me2/3 demethylation, which leads to increased NF-κBp65 activation to support the expression of inflammatory factor monocyte chemoattractant protein-1 (MCP-1) [9]. In addition, Han et al. also demonstrated that EC treated with high glucose exhibited enhanced H3K4me2 and H3K4me3 marks, which caused MCP-1 gene expression via the enrichment of such histone methylation marks on MCP1 promoter [34]. In a separate study, Set7 protein was shown to accumulate in the nucleus in response to hyperglycemia, and such localization activated proinflammatory genes in a Set7-dependent manner [35]. Similarly, in our study, we observed no changes in the expression level of EZH2, while intermittent high glucose caused nuclear localization of EZH2 through its interplay with threonine 367 phosphorylation. Furthermore, nuclear EZH2 assembled PRC2 complex by associating with key PRC2 core proteins; SUZ12 and EED followed by enhancing its recruitment to histone H3. 

The zinc finger transcription factors KLF2 and KLF4 are required for endothelial cell survival to maintain vascular integrity [27]. Endothelial-specific deletion led to embryonic lethality, suggesting that KLF2 in EC is necessary for vascular function [36]. A finding by Atkins et al. (2008) demonstrated that mice lacking one copy of the KLF2 gene exhibited increased atherosclerosis when mated with apoE-deficient mice and fed with a high fat and cholesterol diet [37]. In physiological settings, KLF2 and KLF4 cause inhibition of inflammatory gene expression and upregulation of vascular protective genes [24,25,36]. However, in diabetes, the underlying epigenetic mechanism of KLF2 and KLF4 regulation in EC still remains elusive. Cancer cells exhibited epigenetic silencing of KLF2 through direct transcriptional repression mediated through the EZH2–H3K27me3 axis [38]. A recent finding described the hyper-methylation of DNA in the KLF4 promoter region, thus regulating KLF4 expression in EC [39]. Parallel to this set of observations, our findings indicate that elevated H3K27me3 enriched in the promoter regions of KLF2 and KLF4 in EC upon intermittent high glucose exposure further cause repression of these genes. Blocking EZH2 activity through gene silencing or catalytic inactivation caused reversal of H3K27me3-dependent inhibitory effects on KLF2 in EC exposed to intermittent high glucose.

In ApoE-/-mice, high levels of homocysteine (Hcy) that induced EZH2 expression were detected, which led to H3 at lysine 27 (H3K27) tri-methylation. Global increase in trimethylation of H3K27 was observed in atherosclerotic plaques in the late stage of the pathology [40]. However, the role of EZH2 and its associated catalytic product H3K27me3 in hyperglycemia-induced endothelial dysfunction and apoptosis has not yet been explored. Through the present study, we detected an elevated H3K27me3 level in intermittent high glucose-challenged EC, and reducing the level of H3K27me3 through inhibition or knockdown of EZH2 reversed the intermittent high glucose-dependent inflammatory phenotype of EC. 

In summary, an elevated level of H3K27me3 in EC exposed to intermittent high glucose is responsible for endothelial inflammation. Intermittent high glucose-challenged EC led to an abrupt increase in H3K27me3 through EZH2 nuclear localization that caused repression of KLF2 and KLF4, resulting in the endothelial inflammatory phenotype. Targeting EZH2 caused re-expression of KLF2 and KLF4 and further revoked the inflammatory state of EC imparted through the intermittent high glucose treatment condition. Changes in histone patterning upon hyperglycemia govern an inflammatory switch of EC through the EZH2–H3K27me3 axis, and inhibition of said epigenetic pathway alters the course of the EC inflammatory switch.

## Figures and Tables

**Table 1:** Primer sequences for qPCR analysis of respective transcript or gene promoters.

Gene Name	Sequence of Forward Primer	Sequence of Reverse Primer
*ICAM1* transcript	*5′TTCGTGTCCTGTATGGCCC3′*	*5′CACATTGGAGTCTGCTGGGA3′*
P-Selectin transcript	*5′CCAACCTGCAAAGGCATAGC3′*	*5′GCGTTGCAGCCAAAGTAACA3′*
*VCAM1* transcript	*5′ACGAATGAGGGGACCACATC3′*	*5′TCCAGAGGGCCACTCAAATG3′*
*UTX* transcript	*5′GCAACAGTTAGGTTGGATGC3′*	*5′AGGCATCCTGAACTTTCCCA3′*
*JMJD3* transcript	*5′GGTCTGTTGTACCCCACTGC3′*	*5′CCGCCTCAGTAACAGCCAGA3′*
*KLF2* transcript	5′CGGCAAGACCTACACCAAGA3′	5′TGGTAGGGCTTCTCACCTGT3′
*KLF4* transcript	*5′CCACCTTCTTCACCCCTAGA3′*	*5′AAGGTTTCTCACCTGTGTGG3′*
*GAPDH* transcript	*5′TCGGAGTCAACGGATTTGGT3′*	*5′TTCCCGTTCTCAGCCTTGAC3′*
*KLF2* Promoter Primer	*5′TCCCATCCATCCAGGGTTCT3′*	*5′TCAGAGACTCTCAGGGGAGC3′*
*KLF4* Promoter Primer	*5′TAGAGGGATTCCTGGCGTTG3′*	*5′GATTTTTCCACTCCTCGCCG3′*

## Data Availability

This study includes no data deposited in external repositories.

## Supplementary Materials

The following are available online at www.mdpi.com/article/10.3390/cells10102548/s1, Figure S1: Differential high glucose treatment condition did not alter the viability of cells while specific intermittent high glucose treatment induced ICAM1 expression. Figure S2: Intermittent mannitol treatment that imparts similar level of osmolality as like intermittent high glucose did not alter inflammation associated genes and H3K27me3 in EC. Figure S3: Intermittent hyperglycemia does not alter the expression of Jumonji C domain containing H3K27me3 demethylase UTX and JMJD3. Figure S4: KLF2 and KLF4 downregulation in intermittent hyperglycemia exposed EC. Figure S5: GSK126 exposure to endothelial cells did not affect its viability and specifically caused reduction of H3K27me3. Figure S6: Time dependent reduction in EZH2 protein level in HUVEC transfected with EZH2 siRNA and the effect of siRNA and small molecule combination mediated inhibition of EZH2 on intermittent high glucose dependent reduction in endothelial marker eNOS. Table S1: Table indicate the average Ct values of EZH2 and GAPDH transcript in different treatment sets (EZH2 siRNA transfected cells incubated for 24, 48 and 72 h) including Scrambled siRNA transfected EA.hy926 cells.
