# Supplementary material for: Intermittent High Glucose Elevates Nuclear Localization of EZH2 to Cause H3K27me3-Dependent Repression of KLF2 Leading to Endothelial Inflammation
Source: Cells. 2021 Sep 26;10(10):2548. doi: 10.3390/cells10102548 (PMC8534226; doi:10.3390/cells10102548)
Supplement: Supplementary file 1 [file cells-10-02548-s001.zip › cells-1356817-SI.pdf]

Supplemental data

Ct- Normal glucose (5.5mM)
72- 72 hours high glucose (25mM)
8/16- Three cycles of 8 hours high glucose (25mM) 16 hours normal glucose (5.5mM)
12/12- Three cycles of 12 hours high glucose (25mM) 12 hours normal glucose (5.5mM)
4/68- 4 hour high glucose (25mM) 68 hours normal glucose (5.5mM)
24/48- 24 hour high glucose (25mM) 48 hours normal glucose (5.5mM)

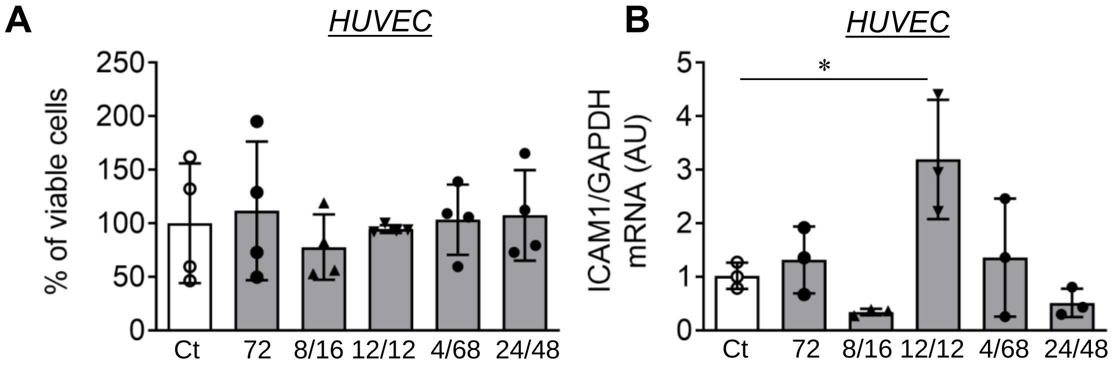

Figure S1. Differential high glucose treatment condition did not alter the viability of cells while specific intermittent high glucose treatment induced ICAM1 expression. (**A**) Cell viability of HUVEC was detected using MTT that are exposed to differential high glucose treatment condition as mentioned in the figure. (**B**) *ICAM1* transcript level was detected in HUVEC challenged with high glucose treatment condition as mentioned in the figure. All analyzed data were normalized to control treatment condition. Values represent the mean ± SD. *$P < 0.05$ by unpaired t test.

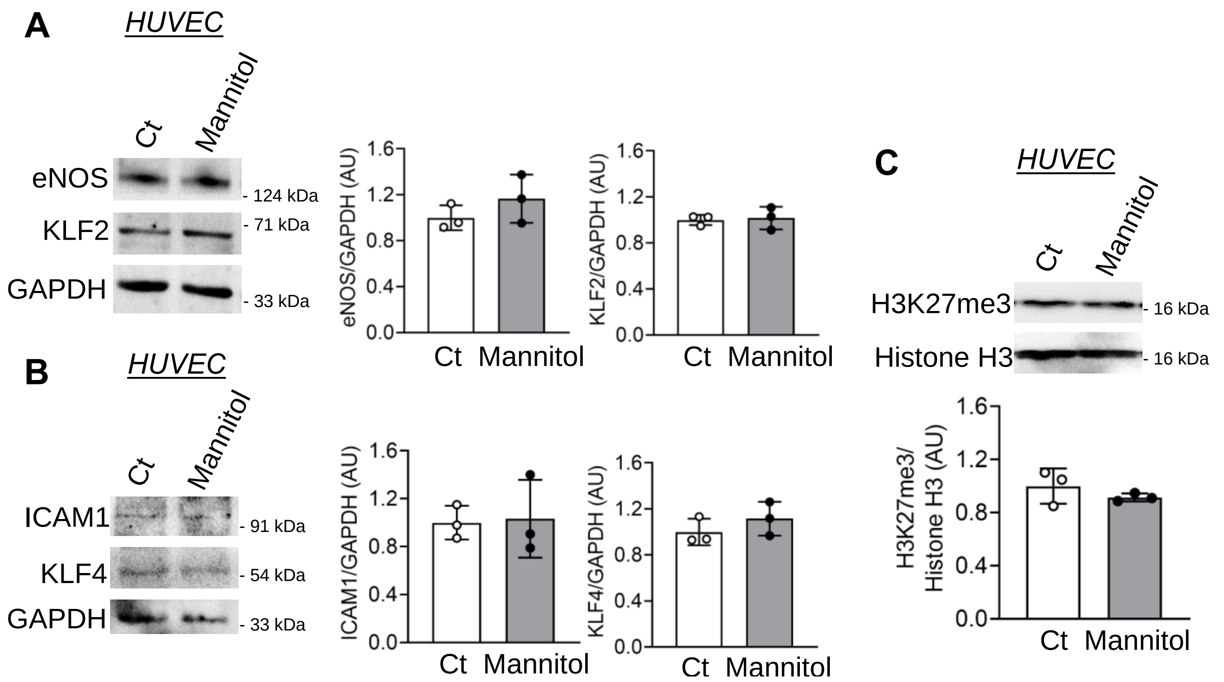

Figure S2. Intermittent mannitol treatment that imparts similar level of osmolality as like intermittent high glucose did not alter inflammation associated genes and H3K27me3 in EC. (**A-B**) Immunoblotting and quantification of eNOS (**A**), KLF2 (**A**), ICAM1 (**B**) and KLF4 (**B**) in HUVEC under exposure to intermittent high glucose (12 hours normal (5.5mM) and 12 hours high glucose (25mM) in three cycles) n=3. (**C**) Immunoblotting and quantification of H3K27me3 (**C**) in HUVEC under exposure to intermittent high glucose (12 hours normal (5mM) and 12 hours high glucose (25mM) in three cycles) (n=3). Values represent the mean ± SD.

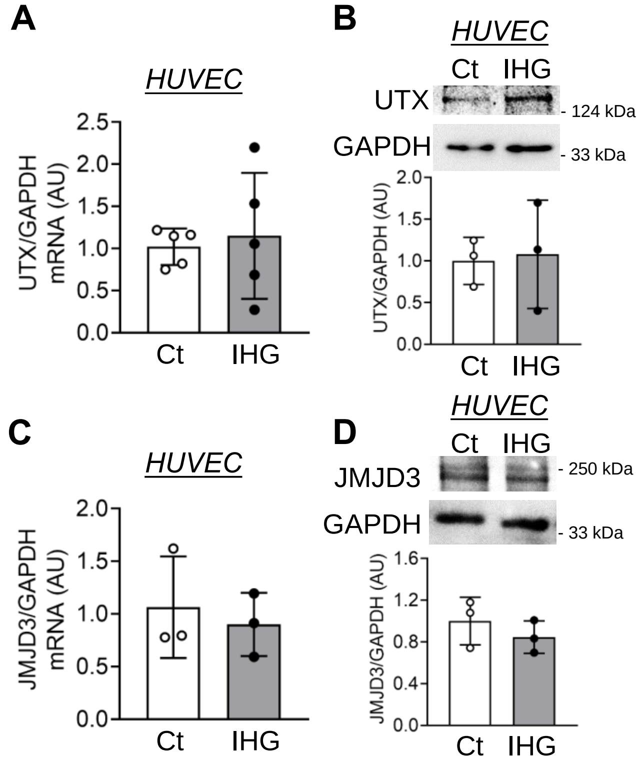

Figure S3. Intermittent hyperglycemia does not alter the expression of Jumonji C domain containing H3K27me3 demethylase UTX and JMJD3. (**A-D**) Real time PCR for UTX transcript (**A**, n=5) and JMJD3 transcript (**C**, n=3) and subsequent immunoblot and quantification of UTX protein (**B**, n=3) and JMJD3 protein (**D**, n=3) in HUVEC treated with intermittent high glucose (alternating 5.5mM and 25mM cycle of 12 hours for three cycles). Values represented as mean ± SD.

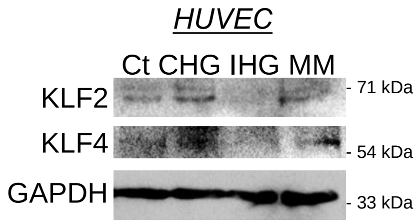

Figure S4. KLF2 and KLF4 downregulation in intermittent hyperglycemia exposed EC. Immunoblotting of KLF2 in HUVEC under exposure to glucose (control, 72 hours high glucose (25mM), 12 hours normal (5.5mM) and 12 hours high glucose (25mM) in three cycles, 4 hours normal glucose (5mM) and 48 hours high glucose (25mM); n=3).

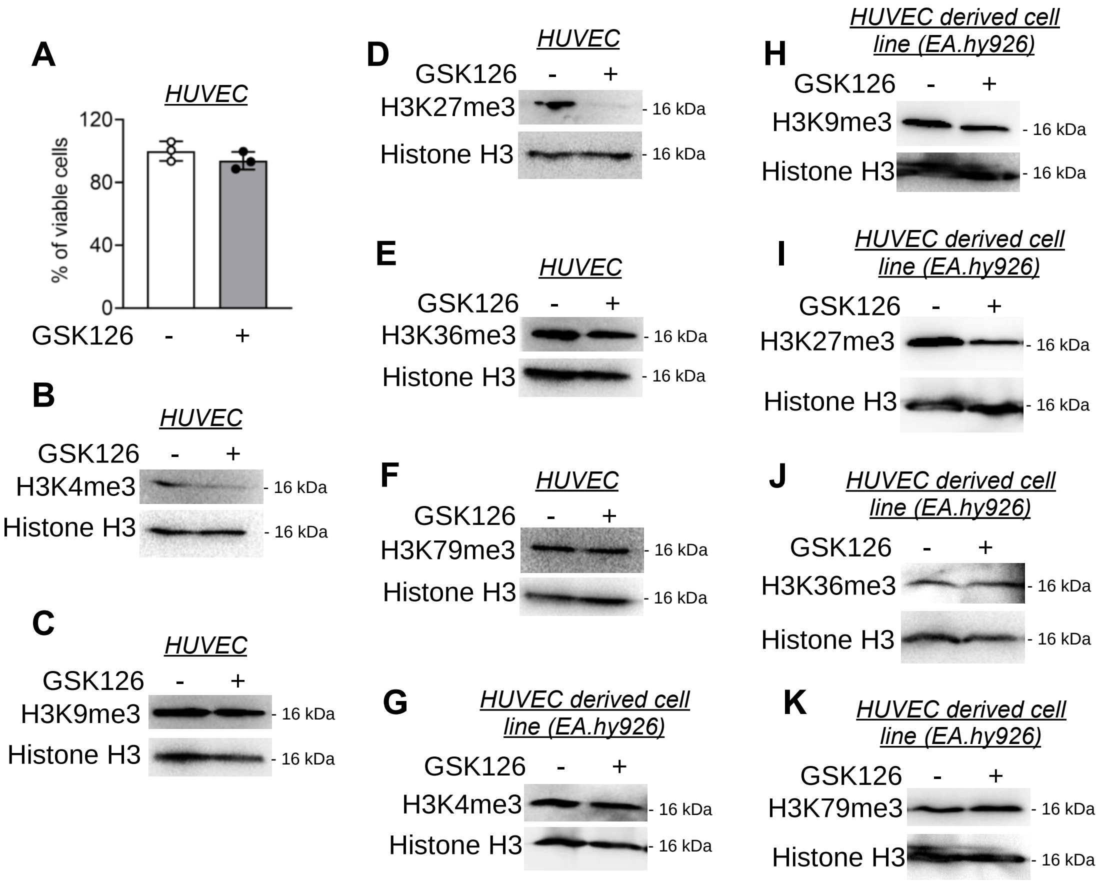

Figure S5. GSK126 exposure to endothelial cells did not affect its viability and specifically caused reduction of H3K27me3. (**A**) Cell viability of HUVEC was detected using MTT that are exposed to GSK126 (10μM) for 72 hours. (**B-F**) Relative level of H3K4me3 (**B**), H3K9me3 (**C**), H3K27me3 (**D**), H3K36me3 (**E**) and H3K79me3 (**F**) were measured in HUVEC treated with GSK126 (10μM) for 72 hours. (**G-K**) Relative level of H3K4me3 (**G**), H3K9me3 (**H**), H3K27me3 (**I**), H3K36me3 (**J**) and H3K79me3 (**K**) were measured in EA.hy926 treated with GSK126 (10μM) for 72 hours. Values represented as mean ± SD.

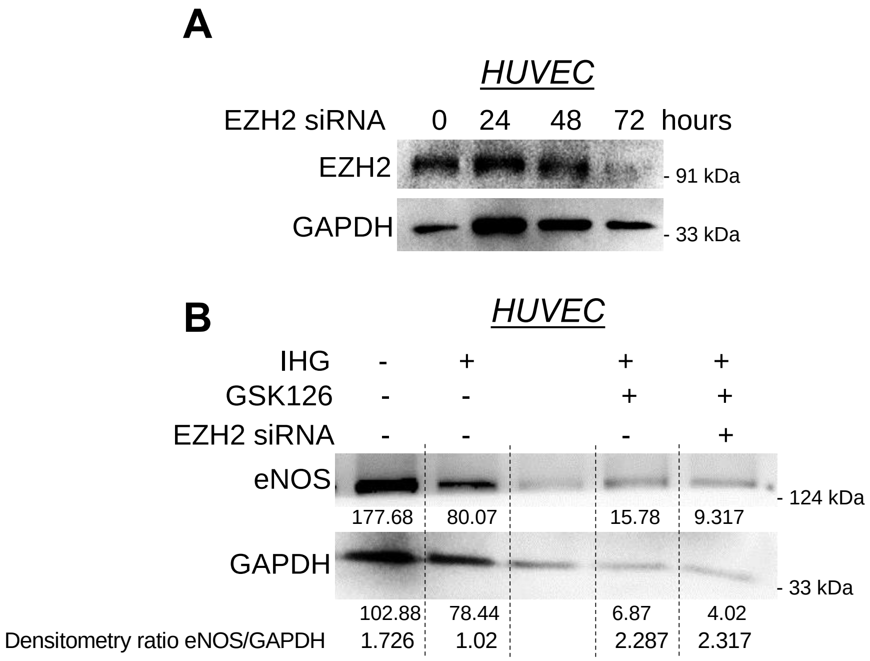

Figure S6. Time dependent reduction in EZH2 protein level in HUVEC transfected with EZH2 siRNA and the effect of siRNA and small molecule combination mediated inhibition of EZH2 on intermittent high glucose dependent reduction in endothelial marker eNOS. (**A**) EZH2 protein level was measured in HUVEC transfected with EZH2 siRNA for 24, 48 and 72 hours. (**B**) EZH2 siRNA transfected HUVEC are treated with GSK126 prior to exposing them to intermittent high glucose treatment condition (12 hours normal (5mM) and 12 hours high glucose (25mM) in three cycles) followed by measuring eNOS protein level.

Treatment Groups	Avg. Ct value of EZH2	Avg. Ct Value of GAPDH	delta Ct
Scr siRNA	31.29	22.7	8.59
EZH2 siRNA (24 hours)	29.36	20.34	9.02
EZH2 siRNA (48 hours)	33.93	23.1	10.83
EZH2 siRNA (72 hours)	Not Amplified	23.3	NA

Table S1. Table indicate the average Ct values of *EZH2* and *GAPDH* transcript in different treatment sets (EZH2 siRNA transfected cells incubated for 24, 48 and 72 hours) including Scrambled siRNA transfected EA.hy926 cells.
